# Comparison of two different methods of establishment of canine urethroplasty model: an experimental trial

**DOI:** 10.1186/s12894-021-00933-1

**Published:** 2021-11-30

**Authors:** Jianpo Zhai, Danhui Zhao, Guanglin Huang, Libo Man, Guoqiang Yan, Chengai Wu

**Affiliations:** 1 Department of Urology, Beijing Jishuitan Hospital, No. 68, Huinanbei Road, Changping District, Beijing, 100096 China; 2 Beijing Research Institute of Traumatology and Orthopaedics, No. 31 Xinjiekou East Street, Xicheng District, Beijing, 100035 China

**Keywords:** Urethroplasty, Animal model, Canine model, Urethral construction

## Abstract

**Background:**

Graft substitute urethroplasty is recommended for patients with long segment anterior urethral stricture. The therapeutic effects of the grafts need to be validated on the animal models. Therefore the aim of this study was to compared the operative time, blood loss, intra- and post- operative complications of two different methods of establishment of canine urethroplasty model.

**Methods:**

Twelve Beagle dogs were randomly separated into control and experimental group using a random number table. Six animals in the control group received the conventional urethroplasty, while the other 6 in the experimental group received the modified procedures. Tube cystostomy and urethroplasty were performed in the control group. The cystostomy not the tube cystostomy were performed in the experimental group, and the testes were simultaneously removed with the scrotum. Per- and postoperative outcomes, complications were evaluated.

**Results:**

The urethroplasty were successfully performed for all dogs and all of these procedures were done by the same surgeon. The median operative time in the control and experimental groups was 186.8 min and 188.7 min respectively. The blood loss in the control and experimental groups was 40.8 ml and 45.8 ml respectively. No intraoperative complications occurred. 3 animals in the control group developed acute urinary retention after the accidental removal of suprapubic bladder tube and the cystostomy was done again. There was no occurrence of urinary retention in the experimental group. 4 animals in the control group developed the perineal hematoma, in which one animal had the urine leakage and incision infection. Perineal hematoma occurred in only one animal in the experimental group.

**Conclusion:**

The occurrence of urinary retention and perineal hematoma decreased in the modified group, in which the cystostomy not the tube cystostomy were performed and the testes with the scrotum were simultaneously removed.

## Background

The treatment of severe urethral stricture has always been a challenge even for skilled urologists [1, 2]. Substitution urethroplasty is recommended for patients with long anterior segmental urethral stricture [3]. Several autologous grafts or flaps from genital and extragenital skin or mucosa have been recommended for patients with anterior urethral stricture [4–7]. Till now, the buccal mucosa is still the most commonly used substitute material. However, the autologous tissues are limited in quantity [8]. Furthermore, significant donor site morbidity has also been reported after its harvesting (16–32% for buccal mucosa grafts), therefore alternative therapeutic options are needed to improve the long-term outcomes [9, 10]. Tissue engineered urethra may overcome some of the aforementioned disadvantages by providing a temporary template to guide the urethral regeneration [11].

The therapeutic effects of the tissue engineered urethra need to be validated on the animal models. Large-animal models, such as the canine model, were preferred because large-animal urethral reconstruction closely mimics the scale and structure of human urethra [12]. In the substitute urethroplasty, the cystostomy has to be done simultaneously to prevent the postoperative urinary retention. The tube cystostomy is the most commonly performed in the conventional surgical procedures [13, 14]. However, the incidence of accidental removal of the cystostomy tube is high in animal studies. Moreover, several other defects of the techniques were still remained, including postoperative urinary retention, urinary leakage, perineal hematoma and incision infection. We currently modified the conventional techniques and compared the safety and effectiveness of two different methods of establishment of canine urethroplasty model.

## Methods

The study was conducted with the approval of the Ethics Committee of Beijing Jishuitan Hospital. 12 Beagle dogs (at an average age of 1.2 y, 10.5 kg in weight), purchased from Beijing Marshall Biotechnology Co. Ltd, were used in the study. The dogs were randomly separated into control and experimental group using a random number table. Six animals in the control group received the conventional urethroplasty, while the other 6 in the experimental group received the modified procedures. The scaffold used in the experimental trial is the decellularized artery.

Dogs were fasted for 12 h and were walked prior to each anesthetic episode and procedure. The animals were sedated with acepromazine (0.05 mg/kg intramuscularly) and anesthesia was induced using a combination of ketamine (5 mg/kg intravenous) and diazepam (0.25 mg/kg intravenous). The animals were intubated and maintained under isoflurane (1–2%) anesthesia. The ventral abdomen from the xiphoid process to the scrotum was clipped, and the prepuce was flushed. A standard preparation scrub was performed on the ventral abdomen with iodophor. The surgical site was isolated using sterile drapes.

### Preparation of scaffold

Carotid arteries were harvested from sacrificed beagle dogs, which were also purchased from Beijing Marshall Biotechnology Co. Ltd, and cut into 4.5-cm-long segments. Decellularized artery was then processed as below. First the carotid arteries were placed in the 0.625% glutaraldehyde solution for fixation for 4 h. Then the glutaraldehyde solution was replaced by a fresh solution of 1.0 M NaOH and 0.5% SDS to remove cells and virus-inactivated was carried out. Thirdly, Decellularized arteries were washed thoroughly by 0.1 M PBS at 4 °C for 48 h to remove residual substances. Finally the processed arteries were aseptically packed after histological examination to determine that it contained no cellular components, and stored at 0–4 °C.

### Cystostomy

**Fig. 1:**
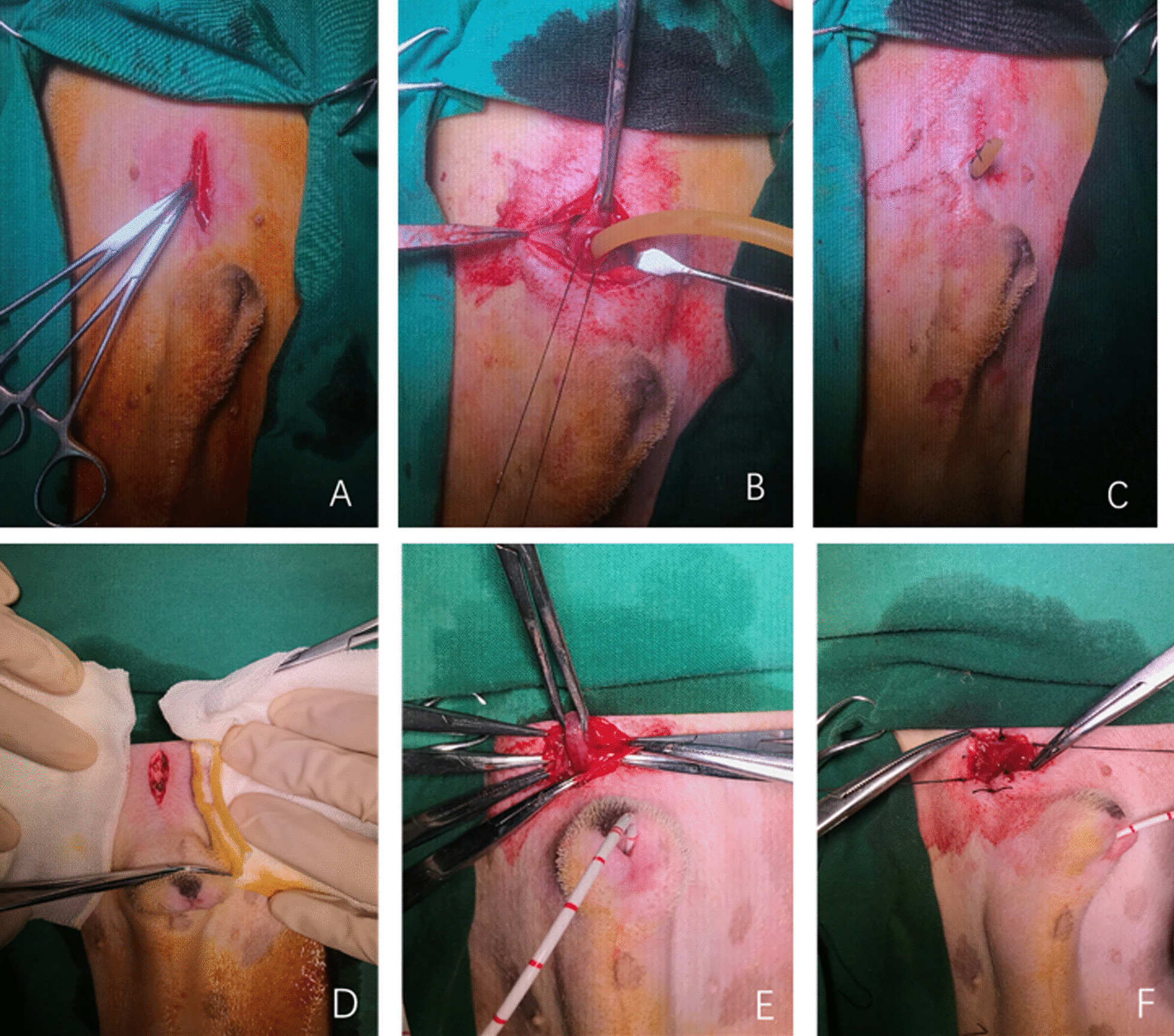
Conventional and modified cystostomy **A** midline incision of lower abdomen. **B** Purse-string suture in the dome of the bladder with a F16 tube inserted into the bladder. **C** Subcutaneous and skin layers closed routinely with the tube secured with a finger-trap suture. **D** Midline incision of lower abdomen. **E** The intended entrance site of bladder pulled out through the incision. **F** The entrance site of the bladder wall incised and 8 interrupted stitches placed around the incision

Conventional tube cystostomy: A midline incision was made in the skin of the lower abdomen (Fig. 1A). The skin, subcutaneous tissue and muscle were incised layer by layer, and the peritoneum was dissected to expose the bladder. A purse string suture was placed in the dome of the bladder, an incision was made within the purse string loop to allow the placement of a 16F tube and then the suture was tightened (Fig. 1B). After confirming that there was no leak around the tube, the bladder was secured to the ventral abdominal wall with four interrupted 3–0 sutures, placed left, right, cranial and caudal to the entrance of the tube into the bladder. The peritoneum and skin was closed with a 3–0 polypropylene suture. The tube was cut off with only 2–3 cm length left outside the body and secured to the skin with a finger-trap suture (Fig. 1C).

Modified cystostomy procedures: the bladder was also exposed through the midline abdominal incision (Fig. 1D). The intended entrance site of bladder was pulled out through the incision with an Allis forceps. The adjacent part of the entrance site was attached to the abdominal wall by interrupted sutures (Fig. 1E). Then the entrance site of the bladder wall was incised and 8 interrupted stitches were placed around the incision. In each stitch, the 3–0 suture passed through the entire thickness of bladder wall, the peritoneum and abdominal wall successively, then the sutures were tightened and tied (Fig. 1F). At last, a F16 tube was inserted into the bladder through the cystostomy and secured with the finger-trap suture.

### Urethroplasty

**Fig. 2:**
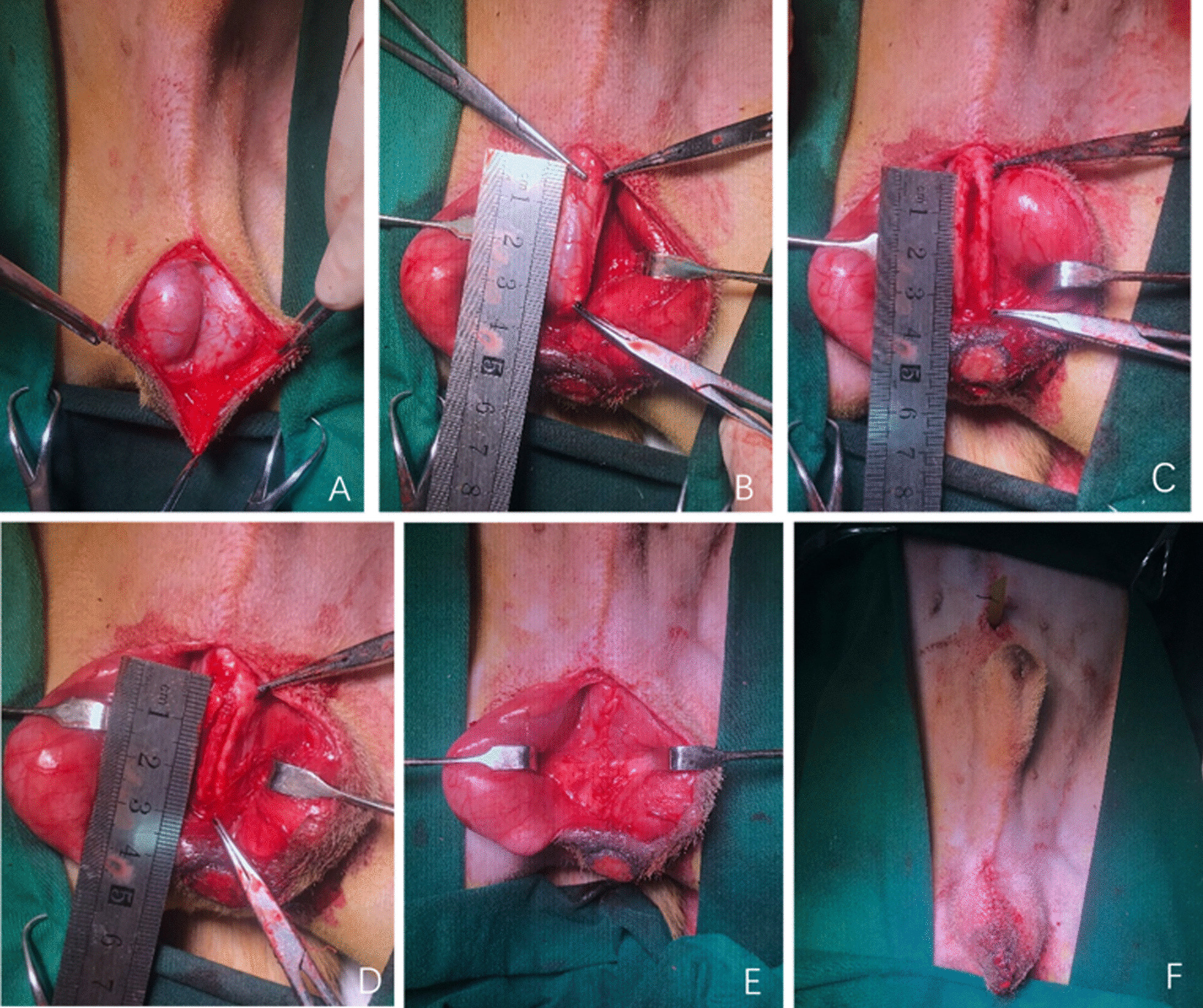
Conventional urethroplasty **A** longitudinal incision over the penoscrotal junction segment of urethra. **B** The corpora cavernosa separated in the midline. **C** A segment of urethra with pre-set length was exposed and dissected. **D** Tubularized scaffold anastomosed to the native ends of the urethra and identification nonabsorbable sutures taken at the distal and proximal ends of the anastomosis. **E** The wound closed in layers. **F** The tube fixed to the external urethral meatus

Conventional urethroplasty procedures: A 6Fr urethral catheter was inserted into the urethra and a longitudinal incision was made over the penoscrotal junction segment of urethra (Fig. 2A). The corpora cavernosa was separated in the midline. In the tubular substitute urethroplasty, a segment of urethra with pre-set length (3 cm) was exposed and dissected (Fig. 2B, C), and the tubularized scaffold was anastomosed with the native ends of the urethral tissues using 5–0 vicryl sutures (Fig. 2D). In the patch substitute urethroplasty, the ventral surface of the urethra was exposed and a segment with pre-set length (3 cm) including half of the urethral circumference was excised. A part of the same length and width of the graft was sutured to the edges of the remaining normal urethra using 5–0 vicryl sutures. Identification nonabsorbable sutures were introduced at the distal and proximal ends of the anastomosis. The wound was closed in layers in a routine fashion (Fig. 2E, F). The 6F urethral catheter was fixed to the external urethral meatus by 3–0 silk suture and the mean duration of the catheter inside the urethra was 14 days.

**Fig. 3:**
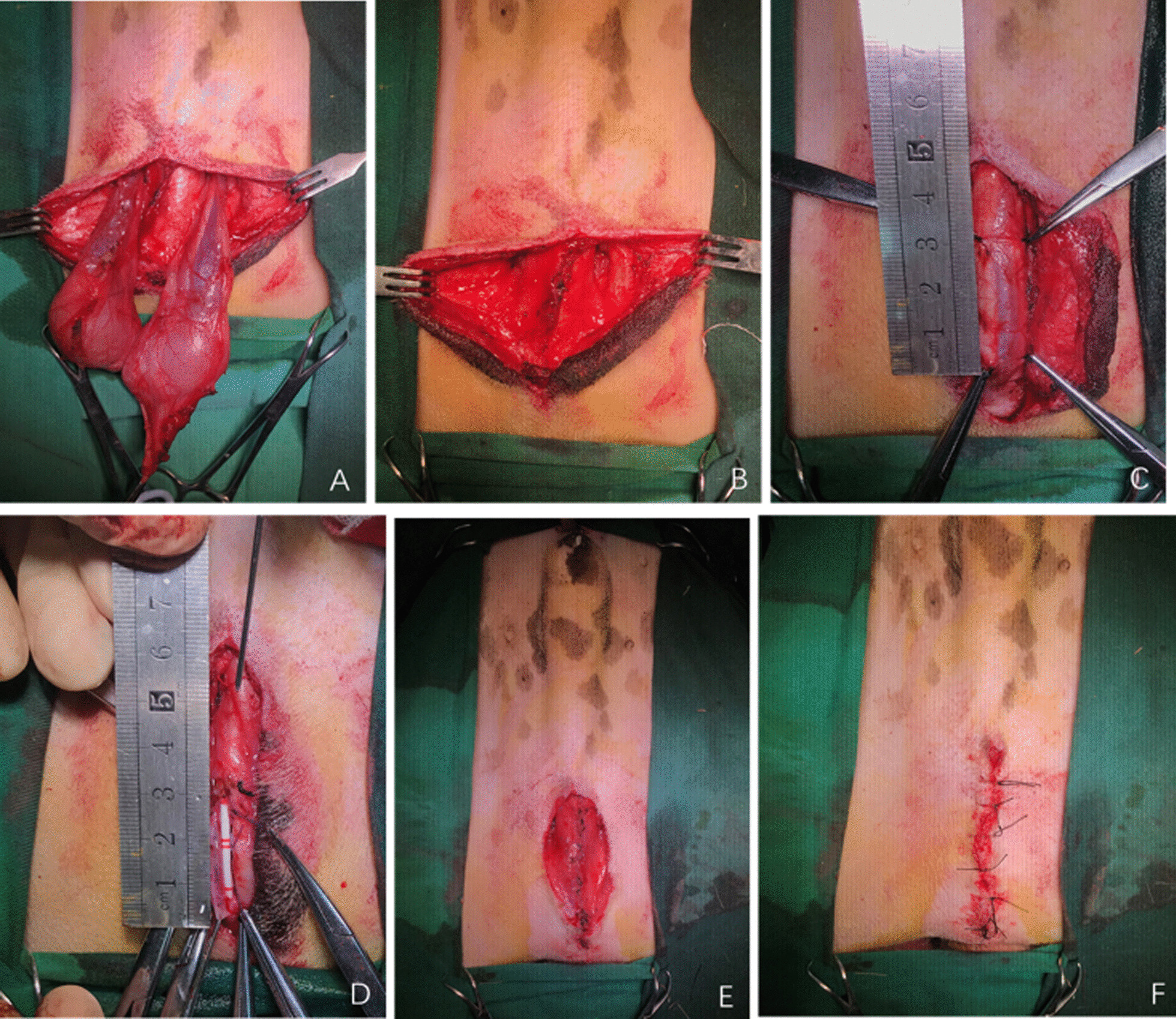
Modified urethroplasty **A** longitudinal incision over the penoscrotal junction segment of urethra. **B** The testes isolated and removed. **C** A segment of urethra with pre-set length was exposed and dissected. **D** Scaffold sutured to the native edges of the urethra. **E** The scrotum and tunica vaginalis removed. **F** The incision closed in layers with interrupted sutures

Modified urethroplasty procedures: the longitudinal incision was also made over the penoscrotal junction segment of urethra after a 6Fr urethral catheter was inserted into the urethra (Fig. 3A). The corpora cavernosa was separated in the midline. Tunica vaginalis on the both sides were incised, and the testes were isolated and removed after the bilateral ligation of the vas deferens (Fig. 3B). Then the substitute urethroplasty was carried out (Fig. 3C, D). After that, the skin of the scrotum and tunica vaginalis were removed (Fig. 3E) and then the incision was closed in layers with interrupted sutures (Fig. 3F).

### Postoperative care

The dogs were housed individually in stainless steel cages. Free access to water and food (Beijing Keaoxieli Feed Co. Ltd.) was provided. The Elizabethan collar was used to prevent the dog from removing the cystostomy tube or urethral catheter. All dogs were given subcutaneous antibiotics (enrofloxacin, 0.1 mg/kg) and analgesic (lornoxicam, 0.27 mg/kg) daily for 3 days. Urethrogram was done 1 month postoperatively and then the experimental animals were humanely killed with intravenous injection of 3–4 ml ketamine. The urethra and bladder were removed from the body and analyzed grossly and histologically.

### Statistical analysis

The Statistical Package for Social Sciences 21.0 software (SPSS 21.0, Chicago, IL, USA) was used for statistical analysis. The characteristics of the two groups were compared with the Mann–Whitney *U* test for continuous variables. The Fisher’s exact or Pearson Chi‐square tests were used for categorical variables. *P* < 0.05 considered as statistically significant.

## Results

### Operative time and blood loss

The urethroplasty were successfully performed for all dogs and all of these procedures were done by the same surgeon. The median operative time in the control and experimental groups was 186.8 min and 188.7 min respectively (*P* = 0.706). The blood loss in the control and experimental groups was 40.8 ml and 45.8 ml respectively (*P* = 0.455) (Table 1).

**Table 1:** Animals’ characteristics and perioperative complications

Variations	Conventional (*N*:6)	Modified (*N*:6)	*P* value
Weight (kg)	8.5 ± 0.3	8.6 ± 0.3	0.807
Operative time (min)	186.8 ± 7.3	188.7 ± 8.9	0.706
Blood loss (ml)	40.8 ± 12.4	45.8 ± 9.7	0.455
Intestinal damage	0	0	
Urinary retention	3	0	0.182
Incision infection	1	0	1.000
Perineal or scrotal hematoma	4	1	0.242
Die	0	0	

### Perioperative complications

All experimental dogs survived to the date of harvest. No intraoperative complications occurred. Three animals in the control group developed acute urinary retention after the accidental removal of suprapubic bladder tube and the cystostomy was done again. There was no occurrence of urinary retention in the experimental group. 4 animals in the control group developed the perineal hematoma, in which one animal had the urine leakage and incision infection. Perineal hematoma occurred in only one animal in the experimental group (Table 1).

## Discussion

Tissue engineering and regenerative medicine studies have led to the development of various bioscaffolds that can be used for urethral repair, such as synthetic materials, collagen, and acellular matrix [15–17]. Results from the study of Orabi and De Filippo demonstrated that collagen scaffolds seeded with cells can be used for longer urethral replacement [12, 18]. Therefore, the scaffolds and seeded-cells are the two critical components of tissue engineering urethra [19–21]. A good blood supply and sterile environment, which can only be provided in good animal models, are essential for the survival both of the scaffolds and the seeded-cells after its transplantation.

It is essential to keep the catheter and tube in place after the urethroplasty. Cystostomy tube can drain the urine out of the body timely, preventing the inflammation caused by the urine leakage [22]. Urethral catheter can provide the support for the substitute grafts, favoring the urethral regeneration [23, 24]. Dogs tend to interfere with the foreign material after surgery, such as all kinds of tubes, dressings, sterile gauzes and so on. Post-operative accidental removal of tubes and catheters can cause the urinary retention and influence the results of the surgery. The occurrence of urinary retention decreased in the modified group. 3 animals in the control group developed the urinary retention, while no animal in the modified group developed. However, the difference was not statistically significant, possibly because of the small sample size. In the tube cystostomy group, it is difficult to insert the cystostomy tube back into the bladder after its accidentally removal, because of the closure of the cystostomy site, therefore cystostomy had to be performed again. While in the modified group, the entrance site of bladder will not closed after the accidental removal of the cystostomy tube and therefore the tube can be easily reinserted into the bladder.

The accidental removal of cystostomy tube or urethral catheter in this study were caused by the bite of the experimental animals. The dogs slipped their neck collars and removed the tubes. In order to avoid the accidental removal of tubes, the neck collars should be firmly worn. Secondly, the tubes or catheters should be secured firmly with the finger-trap suture. Moreover, the exposed portion of the tube should be as short as possible to reduce the interference with the experimental animals.

The occurrence of perineal hematoma decreased in the modified group. 4 animals in the control group and 1 animal in the modified group developed the perineal hematoma. However, these results failed to achieve statistical significance due to the small sample size of the study. In the conventional group, the exudates and bleeding are accumulated in the loose connective tissue due to the elasticity of the scrotum skin, and contributed to the development of the hematoma. While in the modified group, hemostasis can be effectively achieved by local compression that can be easily implemented after the resection of testes and scrotum.

This study provides a good canine model of urethroplasty, in which the occurrence of urinary retention and perineal hematoma were decreased. However, one limitation to this study is the small number of experimental animals. Another limitation is that further investigation is required to clarify the effect of bilateral orchiectomy on the urethral reconstruction.

## Conclusion

The occurrence of urinary retention and perineal hematoma decreased in the modified group, in which the cystostomy not the tube cystostomy were performed and the testes with the scrotum were simultaneously removed.

## Data Availability

The datasets used and/or analysed in the current study is available from the corresponding author on reasonable request.
